# The independent indicators for differentiating renal cell carcinoma from renal angiomyolipoma by contrast-enhanced ultrasound

**DOI:** 10.1186/s12880-020-00436-9

**Published:** 2020-03-30

**Authors:** Hongli Cao, Liang Fang, Lin Chen, Jia Zhan, Xuehong Diao, Yingchun Liu, Chen Lu, Zhengwang Zhang, Yue Chen

**Affiliations:** 1 Department of Ultrasound, Huadong Hospital, Fudan University, 211 West Yan’an Road, Shanghai, China; 2 Shanghai Key Laboratory of Clinical Geriatric Medicine, Shanghai, China; 3 Department of Pathology, Huadong Hospital, Fudan University, Shanghai, China; 4 Department of Urology, Huadong Hospital, Fudan University, Shanghai, China

**Keywords:** Renal cell carcinoma, Angiomyolipoma, Contrast-enhanced ultrasound, Ultrasonography

## Abstract

**Background:**

The value of contrast-enhanced ultrasound (CEUS) in differentiating between renal cell carcinoma (RCC) and angiomyolipoma (AML) was analyzed. The purpose of this study was to identify the independent indicators of CEUS for predicting RCC.

**Methods:**

A total of 172 renal tumors (150 RCCs, 22 AMLs) in 165 patients underwent conventional ultrasound (CUS) and CEUS examinations before radical or partial nephrectomy, and the features on CUS and CEUS were analyzed.

**Results:**

There were significant differences in echogenicity, blood flow signals in color Doppler flow imaging (CDFI), peak intensity, homogeneity of enhancement, wash in, wash out, and perilesional rim-like enhancement between RCC and AML (*P* < 0.05 for all). Multivariate analysis indicated that perilesional rim-like enhancement (*P* = 0.035, odds ratio [OR] = 9.907, 95% confidence interval [CI]: 1.169–83.971) and fast wash out (*P* = 0.001, OR = 9.755, 95%[CI]: 2.497–38.115) were independent indicators for predicting RCC. The area under the receiver operating characteristic (ROC) curve (AUC) for perilesional rim-like enhancement was 0.838 (95% CI: 0.774–0.890) with 76.7% sensitivity and 90.9% specificity, while the AUC of fast wash out was 0.833 (95% CI:0.768–0.885) with 74.7% sensitivity and 81.8% specificity.

**Conclusions:**

This study indicated that CEUS has value in differentiating RCC and AML. Present perilesional rim-like enhancement and fast wash out may be important indicators for predicting RCC.

## Background

Renal cell carcinoma (RCC) originates from the renal tubular epithelium and is one of the most lethal urological malignancies [1, 2]. Its incidence has been increasing year by year, and runs up to 3% of malignant tumors in human beings [3]. Renal angiomyolipoma (AML), comprising 2.0–6.4% of all renal tumors, is the most common benign mesenchymal neoplasm of the renal [4, 5]. Imaging is the main differentiating method for the both above. Up to now, the commonly used imaging diagnostic methods include conventional ultrasound (CUS), computed-tomography (CT), magnetic resonance imaging (MRI), and so on. However, both CT and MRI have disadvantages of high cost, ionizing radiation, and adverse reactions induced by iodine contrast agents or gadolinium contrast agents [6]. Though CUS is non-ionizing, non-invasive, readily available and inexpensive, it is limited in attempting to differentiate RCC from AML [7].

Therefore, a safe and accurate imaging method is needed for differential diagnosing RCC from AML, and microbubble-based contrast-enhanced ultrasound (CEUS) has garnered increasing attention in this field. CEUS has unique advantages with non-ionizing, real-time imaging, and rare and mild adverse reactions induced by contrast agents [8]. The previous studies [9, 10] have reported that CEUS might have a good ability to assess renal masses, but the CEUS characteristics of RCC are still controversial. Xu et al. [11] found that heterogeneous enhancement was a major CEUS characteristic regardless of the subtype of RCC. However, the results of Jiang et al. [12] and Xue et al. [13] showed that for tumors≤3 cm, homogeneous enhancement was more frequently seen on CEUS, regardless of the subtype enhancement.

Thus, the present study aimed at analyzing the diagnostic performance of CEUS in differentiating between RCC and AML proved pathologically, and identifying the independent indicators of CEUS for predicting RCC.

## Methods

### Patients

This was a single-institution retrospective study. This study was approved by the Review Board of Huadong Hospital, and written informed consent was obtained from all patients. Between August 2012 and January 2019, 165 patients with 172 renal masses were recruited for the study, included 145 patients (117 males and 33 females, age range 25–86 years, mean age 61.2 ± 12.4 years) with 150 RCCs and 20 patients (5 males and 17 females, age range 22–75 years, mean age 55.7 ± 16.0 years) with 22 AMLs. Inclusion criteria were as follows: 1) patients underwent CUS and CEUS before radical or partial nephrectomy; 2) renal tumor pathologically confirmed as RCC or AML; 3) sufficient normal renal tissue around mass; and 4) patient had not undergone any invasive treatments before CUS and CEUS. Exclusion criteria were 1) pure cystic mass; 2) the video clips of CEUS were incomplete; and 3) a history of cardiac failure or respiratory disorders.

### CUS and CEUS examination

Both CUS and CEUS were performed by a single radiologist (C.L.) with 17 years of experience in abdominal US and 13 years in CEUS at our institution. The examinations were performed using an ultrasound scanner (4C1 probe, 3–5 MHz, mechanical index < 0.10, Aplio500, Toshiba Medical Systems, Otawara, Japan). Initial CUS was conducted to obtain the position, shape, echogenicity, size, margins, homogeneity, and orientation of the tumor. Then color Doppler flow imaging (CDFI) was used to assess the blood flow of the tumor. Subsequently, optimal section containing both renal lesion and normal adjacent parenchyma was selected, and the ultrasound scanner was switched to CEUS mode. The US contrast agent of SonoVue (Bracco, Milan, Italy), a sulfur hexafluoride (SF6) microbubble stabilized by phospholipids, was used in this study. The freeze-dried powder of SonoVue was shaken with 5.0 ml of normal saline into suspension. According to the weight, height, and age of the patients, a dose of 1.6–2.4 ml of this suspension was individually administered into the antecubital vein in a bolus fashion, followed by a flush of 5.0 ml saline. At the time of contrast agent injection, the keys of the timer and video recorder were pressed simultaneously. Maintain slow shallow breath was required for all patients, and each dynamic contrast image was observed at least 3 min. If a tumor was incompletely assessed, a second injection was repeated 15 min after the first injection. The single images and video clips of CUS and CEUS were stored in the local hard disk for subsequent analysis.

### Imaging interpretation and data evaluation

The images and video clips saved on the local hard disk were independently reviewed in random by two radiologists (D.X.H. and Z.J.), both blinded to the pathological results. Both radiologists had more than 10 years of experience in urinary US and 8 years in reading CEUS images. The CUS characteristics included the mass position, shape, echogenicity, size, margins, homogeneity, orientation, and color flow signals. Referring to the normal renal cortex adjacent to renal mass, the enhancement characteristics of renal mass were analyzed. The CEUS features included the enhancement intensity at peak time, the homogeneity of enhancement, the perilesional rim-like enhancement and the “wash in” and “wash out” mode. The enhancement intensity at peak time was described into hyper-, iso-, and hypo-enhancement. The homogeneity at peak enhancement was classified into homogeneous and heterogeneous. The homogeneous was defined as a renal mass with uniform enhancement, and the heterogeneous was defined as a renal mass with inconsistent enhancement. The perilesional rim-like enhancement, more distinct in the late phase of enhancement, was classified as present or absent. Both the “wash in” and “wash out” of renal masses contrast enhancement were classified as fast, synchronous, or slow. The value of these features was calculated and analyzed to differentiate the diagnosis between RCC and AML. If conclusions of the two radiologists were different, they consulted with a third reviewer to reach a final conclusion by discussions.

### Statistical analysis

Continuous variables were expressed as mean ± standard deviation (SD), and discrete variables as numbers and percentages. The Shapiro-Wilk W test was used to determine whether data was normally distributed, and the F test determine whether the variables had homogeneity of variance. Differences between RCC and AML were analyzed using Independent-Sample t test and Mann-Whitney U test for continuous variables, and Pearson’s chi-square test and Fisher’s exact tests for discrete variables. The variables that showed strong performance in basic statistics were subjected to the multivariate logistic regression analysis for predicting RCC. We calculated odds ratio (OR), 95% confidence intervals (CIs), and *P* value. Those variables with significant and independent influence were isolated and used to fit the receiver operating characteristic (ROC) curve determining the area under the ROC curve (AUC). We determined the optimal cut-off values for the represented indices that showed the highest AUC. The corresponding sensitivity, specificity, and AUC were calculated with 95% CIs. Statistical analysis was performed by using IBM SPSS Statistics version 22.0 for Windows (IBM Corp., Armonk, NY, USA) and MedCalc Statistics version 15.2. A *P* value < 0.05 was considered statistically significant.

## Results

### Characteristics of the enrolled patients

A pathologic diagnosis was obtained for all masses via a laparoscopic or open radical or partial nephrectomy. One single mass was detected in 142 patients with RCCs and 16 patients with AMLs, and two masses were detected in the remaining 7 patients. Of the patients with two nodules, 2 had AML in each kidney, 2 had RCC in each kidney, 1had RCC in the left kidney, 1had RCC and AML in the left kidney, and 1had AML in the left kidney and RCC in the right kidney. Thus, a total of 165 patients with172 renal masses were recruited, 150 (87.2%) were RCCs and 22 (12.8%) were AMLs. The RCCs were clear cell carcinoma (129, 86.0%), papillary carcinoma (11, 7.3%), chromophobe carcinoma (10, 6.7%), respectively. Though age distribution, tumor location, and surgical method were not significantly different between RCCs and AMLs, more percentage of patients with RCC were male than that with AML (*P* = 0.000) (Table 1).

**Table 1:** Patient clinical characteristics

Characteristics	Description	AML(*n* = 22)	RCC(*n* = 150)	*x*^2^ / *t*	*P*-value
Gender	Male	5(22.7)	117(78.0)	28.427	0.000^b^
	Female	17(77.3)	33(22.0)		
Age	mean ± SD (years)	55.7 ± 16.0	61.2 ± 12.4	1.846	0.067^a^
Laterality	Left kidney	12(54.5)	79(52.7)	0.027	0.869^b^
	Right kidney	10(45.5)	71(47.3)		
Tumor location	Upper pole	9(40.9)	45(30.0)	1.403	0.496^b^
	Middle part	5(22.7)	50(33.3)		
	Lower pole	8(36.4)	55(36.7)		
Surgical methods	RN	12(54.5)	111(74.0)	3.564	0.059^b^
	Nephron-sparing PN	10(45.5)	39(26.0)		

*AML* angiomyolipoma, *RCC* renal cell carcinoma, *RN* Radical nephrectomy, *PN* partial nephrectomy, Values are presented as the number (%), ^a^Independent-Sample *t*-test and Mann-Whitney U test, ^b^Pearson’s Chi square test

### CUS characteristics of renal masses

The diameter of RCCs (mean, 40.4 ± 22.1 mm; range, 7-130 mm) and AMLs (mean, 34.7 ± 22.2 cm; range, 9-80 mm) made no significant difference (*P* = 0.258) (Table 2). There were also no significant differences in terms of shape, margin, orientation, and homogeneity between RCCs and AMLs. Significant differences existed in echogenicity and CDFI pattern.

**Table 2:** CUS characteristics of renal masses

Characteristics	Description	AML(*n* = 22)	RCC(*n* = 150)	*x*^2^ / *t*	*P*-value
Size	mean ± SD (mm)	34.7 ± 22.2	40.4 ± 22.1	1.136	0.258^a^
Shape	Round/Oval	20(90.9)	121(80.7)	0.757	0.384^c^
	Irregular	2(9.1)	29(19.3)		
Margins	Well defined	21(95.5)	114(76.0)	3.226	0.072^c^
	Poorly defined	1(4.5)	36(24.0)		
Orientation	Outward from the renal capsule	18(81.8)	101(67.3)	1.888	0.169^b^
	Inward at the renal parenchyma	4(18.2)	49(32.7)		
Echogenicity	Hyper-echoic	17(77.3)	23(15.3)	33.153	0.000^b^
	Iso-echoic	1(4.5)	28(18.7)		
	Hypo-echoic	4(18.2)	99(66.0)		
Homogeneity	Homogeneous	17(77.3)	97(64.7)	1.364	0.243^b^
	Heterogeneous	5(22.7)	53(35.3)		
Blood flow signals in CDFI	Abundant inside	1(4.5)	25(16.7)	25.451	0.000^d^
	Inside and perilesional	0(0)	37(24.7)		
	Perilesional	0(0)	29(19.3)		
	Slight inside	5(22.7)	10(6.7)		
	Without	16(72.7)	49(32.7)		

*AML* angiomyolipoma, *RCC* renal cell carcinoma, *CDFI* color Doppler flow imaging; Values are presented as the number (%); ^a^Independent-Sample *t*-test and Mann-Whitney U test; ^b^Pearson’s Chi square test; ^c^Continuous Correction Chi square; ^d^Fisher’s exact test

### CEUS characteristics of renal masses

CEUS characteristics of renal masses are listed in Table 3. All the indices (peak intensity, homogeneity, wash in, wash out, and perilesional rim-like enhancement) are significantly different between RCCs and AMLs (*P* < 0.05 for all).

**Table 3:** CEUS characteristics of renal masses

Characteristics	Description	AML(n-22)	RCC(*n* = 150)	*x*^2^	*P*-value
Enhancement intensity	Hyper-enhancement	4(18.2)	112(74.7)	27.175	0.000^b^
	Iso-enhancement	10(45.5)	17(11.3)		
	Hypo-enhancement	8(36.4)	21(14.0)		
Homogeneity	Homogeneous	17(77.3)	72(48.0)	6.584	0.010^a^
	Heterogeneous	5(22.7)	78(52.0)		
Wash in	Fast	2(9.1)	73(48.7)	17.642	0.000^a^
	Synchronous	11(50.0)	59(39.3)		
	Slow	9(40.9)	18(12.0)		
Wash out	Fast	1(4.5)	84(56.0)	34.841	0.000^a^
	Synchronous	2(9.1)	30(20.0)		
	Slow	19(86.4)	36(24.0)		
perilesional rim-like enhancement	Present	2(9.1)	115(76.7)	40.279	0.000^a^
	Absent	20(90.9)	35(23.3)		

*AML* angiomyolipoma, *RCC* renal cell carcinoma; Values are presented as the number (%); ^a^Pearson’s Chi square test; ^b^Fisher’s exact test

### The independent indicators correlated with RCCs

**Fig. 1:**
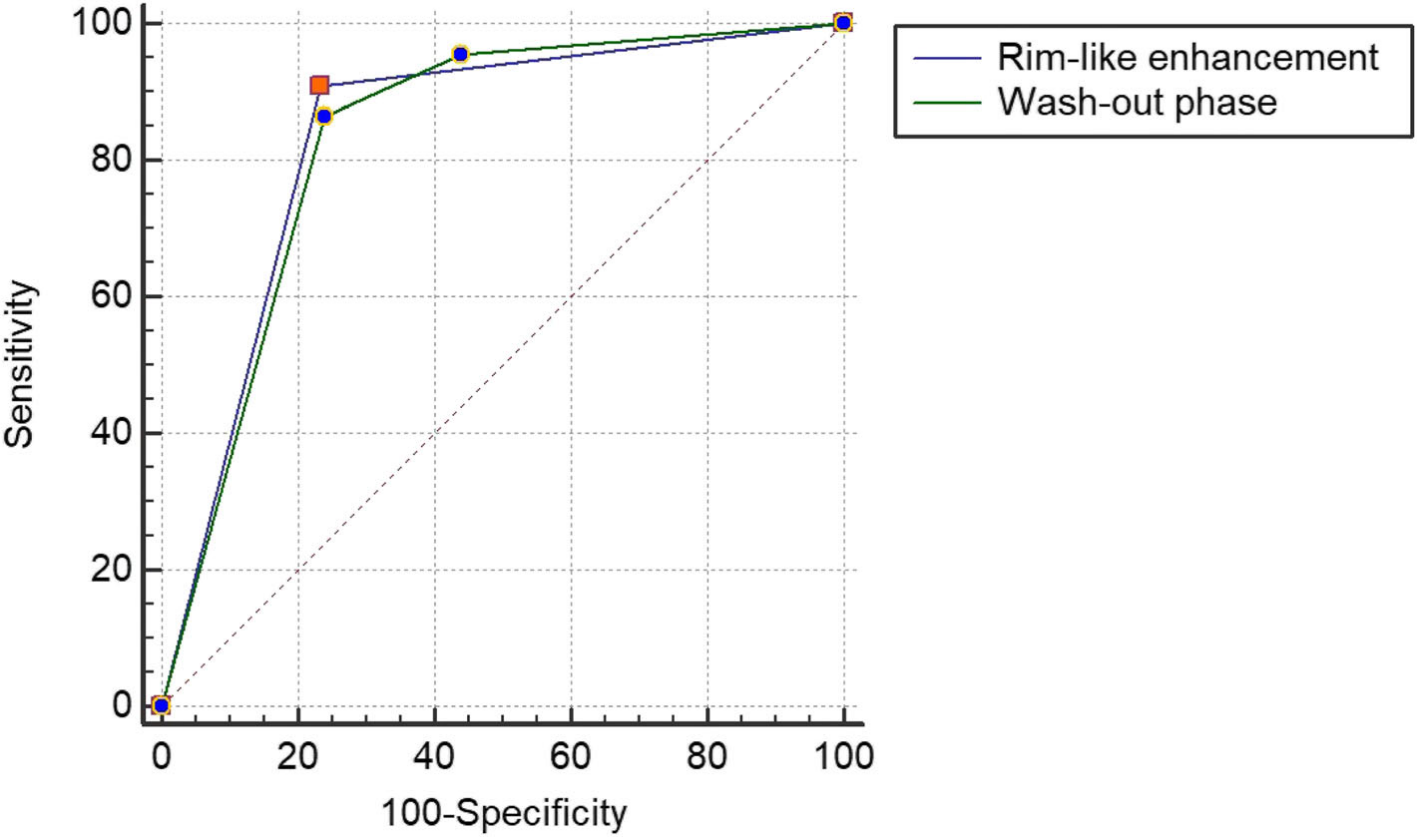
Receiver operating characteristic (ROC) curve demonstrated sensitivities and specificities of significant indicators of renal cell carcinoma. The areas under the curves were 0.838 and 0. 833 for perilesional rim-like enhancement and wash out, respectively

Multivariate analysis was used to identify the potential indicators of RCCs. The results showed that perilesional rim-like enhancement and fast wash out were independent indicators correlated with RCCs (Table 4). Figure 1 shows the ROC curves of perilesional rim-like enhancement and fast wash out correlated with RCCs. The AUC of perilesional rim-like enhancement was 0.838 (95% CI:0.774–0.890) with 76.7% sensitivity and 90.9% specificity, while the AUC of fast wash out was 0.833 (95% CI: 0.768–0.885) with 74.7% sensitivity and 81.8% specificity (Table 5).

**Table 4:** Multivariate analysis with variable selection for predicting RCCs

Characteristics	B	SE	OR(95%CIs)	*P*-value
Echogenicity	0.949	0.535	2.583(0.905–7.369)	0.076
Blood flow signals in CDFI	0.498	0.463	1.645(0.663–4.078)	0.283
Enhancement intensity	0.194	0.798	1.214(0.254–5.802)	0.808
Homogeneity	0.475	1.035	1.608(0.211–12.233)	0.646
Wash in	1.433	0.883	4.191(0.742–23.669)	0.105
Wash out	2.278	0.695	9.755(2.497–38.115)	0.001
perilesional rim-like enhancement	2.293	1.090	9.907(1.169–83.971)	0.035

*RCC* renal cell carcinoma, *B* regression coefficient, *SE* standard error; *OR (95%CIs)* odds ratio (95% confidence intervals)

**Table 5:** ROC analyses of the independent variables for predicting RCCs

Variables	Cut-off value	Sensitivity (%)	Specificity (%)	AUC (95%CIs)
Perilesional rim-like enhancement	Present	76.7	90.9	0.838(0.774–0.890)
Wash out	Fast	74.7	81.8	0.833(0.768–0.885)

*RCC* renal cell carcinoma, *AUC* area under the curve, *95% CIs* 95% confidence intervals

## Discussions

RCC, the most common renal malignancy, is characterized by numerous thin-walled blood vessels with rich blood flow, and common findings include intra-tumoral necrosis, hemorrhage, and calcification [14]. AML, the most common renal benign neoplasm, contains varying proportion of thick-walled blood vessels, smooth muscle, and fat tissue [4]. Most AMLs need only active surveillance rather than invasive treatment, but for RCC, especially for clear cell RCC, surgical resection is the preferred therapy [15, 16]. Therefore, it is important to differentiate them for the prognostic evaluation and clinical treatment decision.

Compared to CT and MRI, CUS is usually the preferred choice for detecting renal lesions because it is readily available, inexpensive, noninvasive, non-ionizing, and provides images in real time [17]. However, it has limited use when attempting to differentiate between RCC and AML because of its lower accuracy in the characterization of some renal masses [7]. To the extent known, hypoechoic renal masses are mostly considered to be malignant while hyperechoic and iso-echoic renal masses are often referred to as benign. The current study showed that, among all the characteristics on CUS, there was significantly different in term of echogenicity between RCC and AML (*P* = 0.000). However, hyperechoic RCCs were noted in 23 masses (15.3%) and iso-echoic RCCs were noted in 28 masses (18.7%). Four AMLs (18.2%) were hypoechoic and one (4.5%) was iso-echoic on CUS in this study. Therefore, CUS had limited ability to distinguish between RCC and AML. As for CDFI, RCC differed from AML with respect to blood flow signals (*p* = 0.000). However, there were 16(72.7%) AMLs and 49(32.7%) RCCs without blood flow signals in the study. Due to overlap in imaging features between some cases of AML and RCC, additional imaging to further characterize renal lesions is recommended.

The previous studies have reported that CEUS imaging technology, without severe risk or discomfort, has a good ability to assess renal lesions [18–20], but the CEUS characteristics of RCC are still controversial. Thus, the present study aimed at analyzing the diagnostic performance of CEUS in differentiating between RCC and AML proved pathologically. And our study showed that perilesional rim-like enhancement and fast wash out were independent indicators correlated with RCC.

**Fig. 2:**
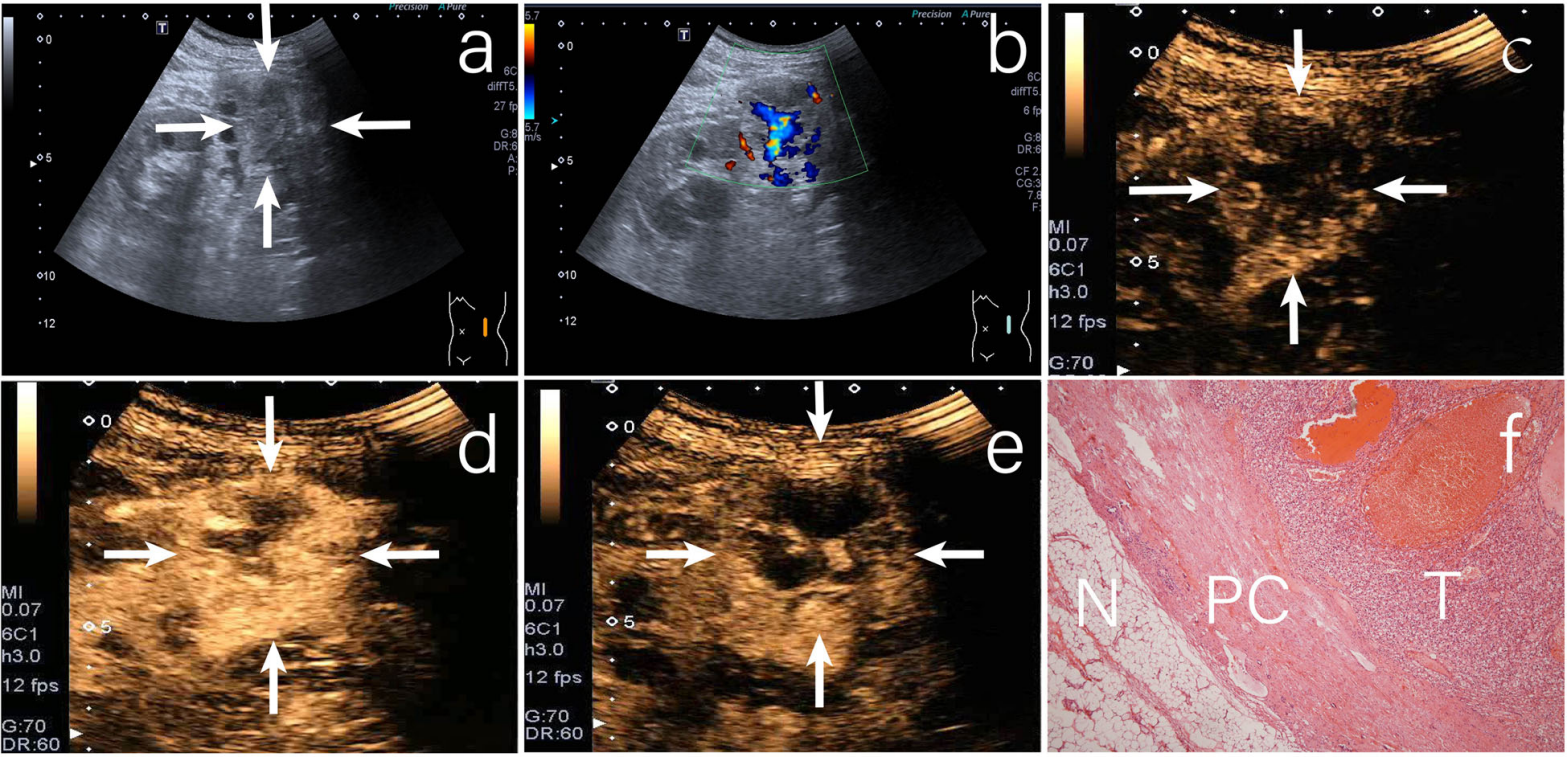
A case of clear cell renal carcinoma. **a** CUS revealed a hypo-echoic renal mass located in the middle of the left kidney (arrows); **b** CDFI revealed rich blood flow signals in the tumor; **c** CEUS imaging in the early phase showed fast wash in at the region of the tumor. Peritumoral rim-like enhancement was observed (arrows); **d** CEUS imaging at peak enhancement revealed heterogeneous hyperenhancement (arrows); **e** CEUS imaging in the late phase showed fast wash out in the region of the tumor (arrows); **f** Photomicrograph showed tumor pseudocapsule (PC) of compressed renal parenchyma and fibrous tissue between tumor (T) and normal kidney (N). (hematoxylin and eosin stain, original magnification 100×)

The perilesional rim-like enhancement around the tumor was considered to represent the presence of a pseudocapsule (Fig. 2). It results from tumor growth producing compression, ischemia, and necrosis of adjacent normal parenchyma, with subsequent deposition of fibrous tissue [16, 21]. In our study, a pseudocapsule was observed on the histologic examination. And in this study, CEUS showed that more percentage of perilesional rim-like enhancement was observed in RCC than in AML (*p* = 0.000). The perilesional rim-like enhancement was observed in 76.7% (115/150) of RCCs, which was similar to the figures reported by Xu et al. [11] and Van et al. [22]. The presence of perilesional rim-like enhancement was regarded as an important predictor of RCC. Additionlly, the current study found that 2(9.1%) AMLs presented with incomplete rim-like enhancement. This might be related to the distribution of blood vessels in AML.

The wash out would be possible in arteriovenous fistulas or in normal physiological reflux through the renal vein [23]. Compared with the normal renal parenchyma, RCCs often exhibit fast wash out, while AMLs more often show slow wash out [24]. Xue et al. [13] assessed the difference of enhancement patterns among the three RCC subtypes with CEUS, their results demonstrated that most RCCs were fast wash out with the percentage of 47.5(95/200), 89.7(52/58), 76.5(39/51) for clear cell RCCs, papillary RCCs, chromophobe RCCs, respectively. Our results showed that 56.0% (84/150) RCCs exhibited fast wash out. This may be related to the proportion of RCC subtypes included in this study. Sun et al. [25] reported that 26%(5/19) of AMLs exhibited fast wash out, while, 74% lesions were confirmed by two enhanced imaging examinations including CEUS and CT/MRI. In our study, all the lesions were diagnosed via surgical pathology and only 4.5% (1/22) of AMLs exhibited fast wash out.

**Fig. 3:**
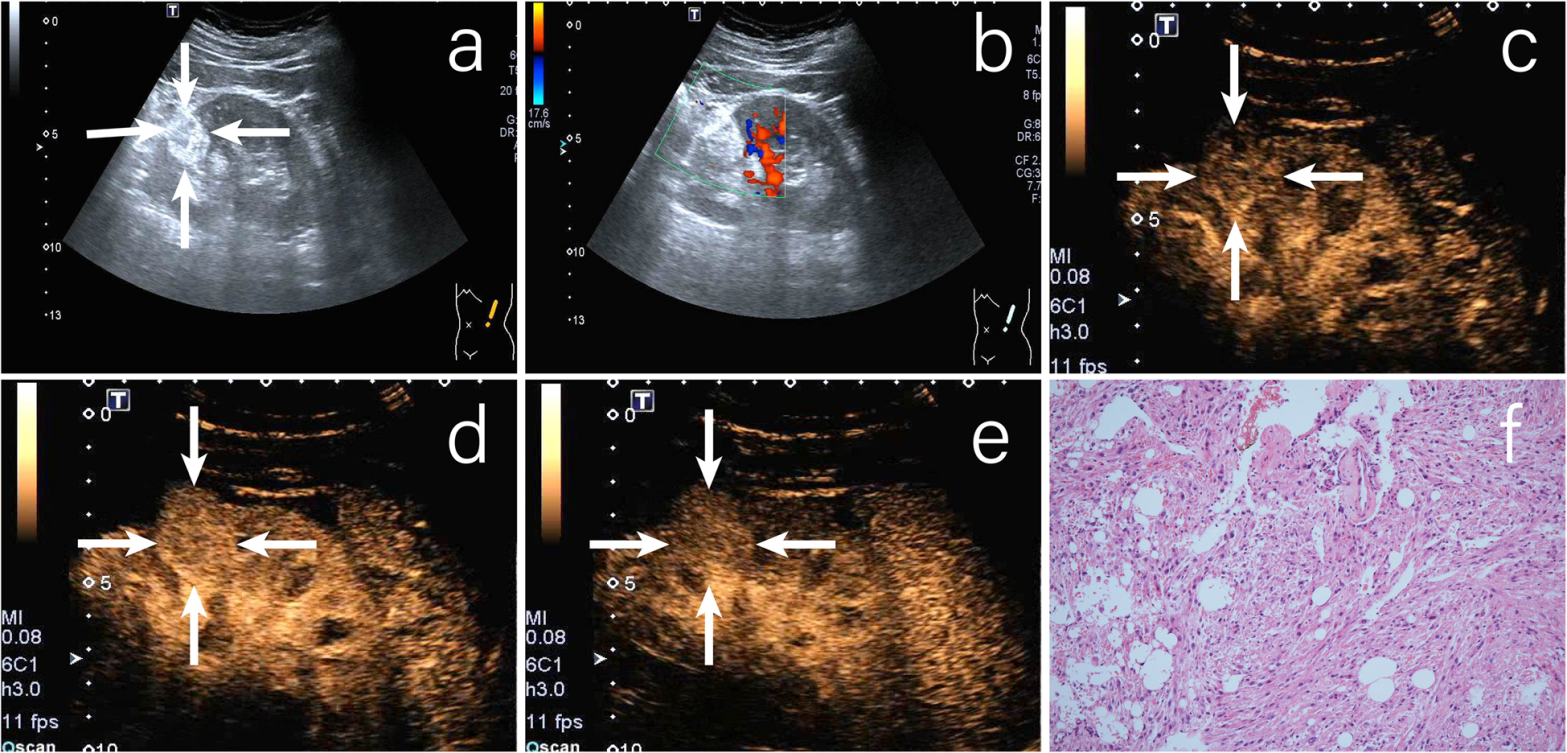
A case of renal angiomyolipoma. **a** CUS revealed a hyper-echoic renal mass located in the lower pole of the right kidney (arrows); **b** CDFI revealed that there was no blood flow signal in the tumor; **c** CEUS imaging in the early phase showed slow wash in at the region of the tumor (arrows); **d** CEUS imaging at peak enhancement revealed homogeneous hypo-enhancement (arrows); **e** CEUS imaging in the late phase showed slow wash out in the region of the tumor (arrows); **f** Photomicrograph showed renal angiomyolipoma which contains varying proportion of thick-walled blood vessels, smooth muscle, and fat tissue. (hematoxylin and eosin stain, original magnification 100×)

Heterogeneous enhancement on CEUS correlated with the existence of hemorrhage, necrosis and cystic change [26]. Though, RCCs were manifested mostly as heterogeneous enhancement as a result of the rapid growth of the tumor and proneness to ischemic necrosis, heterogeneous enhancement was not the independent indicators for predicting RCCs in our study. This may be related to the size of the tumors included in this study. Moreover, Jiang et al. [12], Xue et al. [13] and Xu et al. [27] analyzed the CEUS characteristics of RCC in relation to tumor size. Their studies showed that heterogeneous enhancement was mainly seen in tumors> 3 cm (72.9–91%), compared to 28.2–55% in tumors≤3 cm. This might because that the small tumors grow slowly and rarely have necrosis change. Though most AMLs are discovered incidentally, some AMLs can present with spontaneous hemorrhage, particularly in masses larger than 4 cm [28]. Moreover, Lu et al. [29] reported that 17.6% (6/34) AMLs showed heterogeneous enhancement. In this study, 22.7% (5/22) AMLs showed heterogeneous enhancement (Fig. 3), and all the masses were larger than 4 cm.

The main limitation of our study is the relatively small number of AMLs (*n* = 22) cases, so the possibility of selection bias should be considered. Prospective studies with larger numbers of AMLs patients are required to validate our results. An additional limitation is that we restricted our analysis to RCCs and AMLs, without considering its subtypes, other benign tumors, or other malignant tumors. Further studies should be performed for the differentiation of all the above. A further limitation is that pathologic findings proven by surgical resection as an inclusive criterion exclude already characterized nonsurgical masses, such as small typical AMLs.

## Conclusion

Our findings suggest that CEUS imaging features including wash out and perilesional rim-like enhancement may be important indicators for predicting RCCs. These imaging features may help differentiate RCCs for the prognostic evaluation and clinical treatment decision.

## Data Availability

Data is available upon request from the corresponding author.

## Abbreviations

AML: Angiomyolipoma
AUC: Area under the curve
B: Regression coefficient
CDFI: Color Doppler flow imaging
CEUS: Contrast-enhanced ultrasound
CI: Confidence interval
CUS: Conventional ultrasound
OR: Odds ratio
PN: Partial nephrectomy
RCC: Renal cell carcinoma
RN: Radical nephrectomy
ROC: Receiver operating characteristic
SE: Standard error
